# Power enhancement of heat engines via correlated thermalization in a three-level “working fluid”

**DOI:** 10.1038/srep14413

**Published:** 2015-09-23

**Authors:** David Gelbwaser-Klimovsky, Wolfgang Niedenzu, Paul Brumer, Gershon Kurizki

**Affiliations:** 1 Department of Chemistry and Chemical Biology, Harvard University, Cambridge, MA 02138, USA; 2 Department of Chemical Physics, Weizmann Institute of Science, Rehovot 7610001, Israel; 3 Chemical Physics Theory Group, Department of Chemistry and Centre for Quantum Information and Quantum Control, University of Toronto, Ontario M5S 3H6, Canada

## Abstract

We explore means of maximizing the power output of a heat engine based on a periodically-driven quantum system that is constantly coupled to hot and cold baths. It is shown that the maximal power output of such a heat engine whose “working fluid” is a degenerate *V*-type three-level system is that generated by two independent two-level systems. Hence, level degeneracy is a thermodynamic resource that may effectively double the power output. The efficiency, however, is not affected. We find that coherence is not an essential asset in such multilevel-based heat engines. The existence of two thermalization pathways sharing a common ground state suffices for power enhancement.

The rapport between quantum mechanics and thermodynamics is still an open problem^1^,^2^. Its technological and fundamental implications have motivated numerous proposals of heat engines based on quantum systems^3^,^4^,^5^,^6^,^7^,^8^,^9^,^10^,^11^,^12^,^13^,^14^,^15^,^16^,^17^,^18^,^19^,^20^,^21^,^22^. Two main issues underlie such proposals: What are the bounds on the performance of quantum heat engines, i.e., their power output and efficiency^1^,^2^,^23^,^24^,^25^,^26^, and what thermodynamic properties (or resources) of quantum systems determine these bounds^27^,^28^,^29^,^30^,^31^? A pioneering approach addressing these issues^32^,^33^ has suggested that steady-state coherence^34^,^35^,^36^ between the levels of a quantum system is a thermodynamic resource.

Here we wish to elucidate these issues from first principles. To this end we resort to a fully solvable model of a steady-state, continuous-cycle, heat engine that is based on a periodically-driven quantum system (“working fluid”) constantly coupled to hot and cold baths^15^,^21^. Consistency with the first and second laws of thermodynamics is enforced in this theory by the construction of appropriate heat currents flowing between the baths via the system^21^,^37^.

To account for the possible rôle of coherences we extend this theory, hitherto applied to a two-level system (TLS) working fluid^15^,^21^, to an analogous heat engine based on a *V*-type three-level system as depicted in Fig. 1a. We have chosen a *V*-system for being the simplest working fluid wherein *coherences* may persist at steady state, and possibly affect the engine performance. The performance of an engine based on such a *V*-system is compared to a TLS-based heat engine (cf. Fig. 1b), where steady-state coherence is absent. We show that the power output of the *V*-system may be boosted by up to a factor of 2 compared to its TLS counterpart. This boost is associated with correlations that arise between the possible thermalization channels in the *V*-system that constitute a hitherto unexploited thermodynamic resource. Such correlations exist even in the absence of coherence, because the degenerate excited states exchange populations with each other via their *common* ground state. However, steady-state coherence does not affect the efficiency, nor does maximal power boost necessarily require coherence, since thermalization correlations may be incoherent.

## Qubit-based heat machine revisited

A continuous-cycle quantum heat machine based on a single qubit (TLS) as working fluid has been studied in ref. 21. This TLS is simultaneously and permanently coupled to cold and hot heat baths, while its transition energy is periodically modulated by some external field according to the Hamiltonian


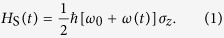


This external field plays the rôle of a piston and allows for work extraction or supply. The dipolar coupling of the atom to the cold and hot baths in the rotating-wave approximation reads^38^


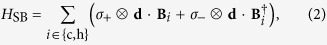


with the transition-dipole moment **d** and the Pauli operator 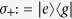 describing the excitation of the atom and its adjoint 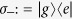 describing de-excitation.

As detailed in^37^ the periodicity of the modulation implies that the dynamics of the system’s density matrix in the interaction picture is governed by a linear combination of “sub-bath” Lindblad operators, i.e., operators associated with the two baths *i* ∈ {c, h}, evaluated at the harmonic (Floquet) sidebands *q* = 0, ±1, ±2,… of the modulation frequency Ω. The master equation in the weak-coupling limit then reads


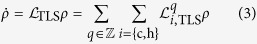


with the Liouvillian superoperators of the (*i*, *q*) “sub-baths”


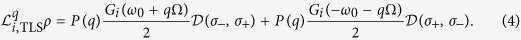


Here *P*(*q*) is the weight of the *q*th harmonic (determined by the modulation form)^37^ and the dissipator reads 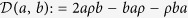 for any system operators *a*, *b*. The factors *G*_*i*_(±*ω*) are the coupling spectra to the *i*th bath and depend on the bath autocorrelation functions 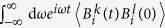, where 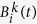 denotes the *k*th component of **B**_*i*_(*t*) in the interaction picture. These spectra fulfill the KMS condition^39^ 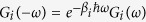, where for a bosonic bath, 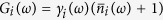, *γ*_*i*_(*ω*) being the frequency-dependent transition rate induced by the *i*th bath and 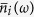 denoting the corresponding number of thermal quanta at inverse temperature *β*_*i*_ = 1/*k*_B_*T*_*i*_.

The heat currents between the cold and the hot baths and the TLS evaluate to^21^


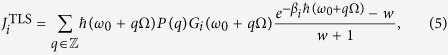


and the power (time derivative of the work) according to the first law, reads


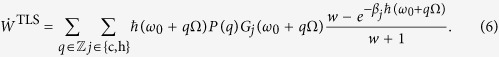


Here 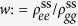 is the ratio between the excited- and the ground-state steady-state populations of the qubit. We here follow the convention that negative power means work extraction (operation as an engine).

This conceptually simple heat machine can be operated “on demand” as a heat engine (the extracted work is manifested by a coherent amplification of the external field) or as a refrigerator, depending on the modulation rate Ω. The machine behaves as an engine if the rate is below some critical value, whereas above this value it acts as a refrigerator^21^.

A detailed analysis of the heat currents (5) and the power (6) reveals that at the critical rate the switch-over from the engine to the refrigeration mode ensures compatibility with the second law—this is precisely the rate at which the engine reaches Carnot efficiency and yields vanishing power. Strikingly, the engine’s efficiency at maximum power can surpass the Curzon–Ahlborn efficiency^40^ under certain conditions on the bath spectra^21^.

This heat machine operates at the steady-state (limit cycle) of the corresponding dissipative time evolution of the working fluid. Naturally, coherence is absent in the system’s steady state. In order to study the effects of coherences, we now extend this TLS-based model to a degenerate three-level system.

### Steady-state treatment of *V*-system heat machines

We consider a *V*-type three-level system with degenerate excited states 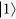 and 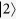, ground state 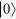 and transition frequency *ω*_0_. To operate a heat machine, we simultaneously connect this system to two (hot and cold) baths, which induce transitions 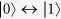 and 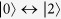. The “piston” periodically modulates both excited states^21^, which results in the same periodic transition frequency *ω*_0_ + *ω*(*t*) as for a TLS (see Eq. 1), with 
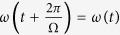
, where Ω denotes the modulation rate. The dipolar system–bath interaction is described by the following generic Hamiltonian [a generalization of the case presented in ref. 39 and in Eq. (2)] in the rotating-wave approximation,


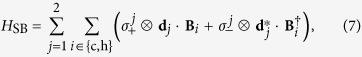


where 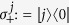 and 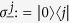 are the excitation (de-excitation) Pauli operators for the *j*th transition, **d**_*j*_ is the transition dipole between the excited state 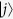 and the ground state 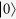, and **B**_*i*_ is the hot (h) or cold (c) bath operator. For simplicity we here restrict the treatment to real dipoles of equal strength, 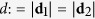. These transition dipoles need not be parallel (aligned), as discussed below.

Based on the interaction Hamiltonian (7), the Floquet-expanded master equation in the weak-coupling limit has the same form as (3),


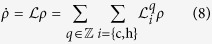


but the Liouvillian superoperators for the *degenerate V*-type three-level system, coupled to the (*i*, *q*) “sub-baths” are now generalizations of (4) (see supplemental material),


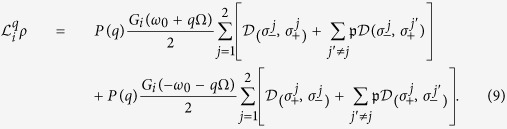


Here the dissipators 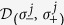 and 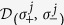 describe emission and absorption involving separate transitions (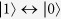 and 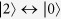) via their common ground state, and hence population transfer between 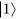 and 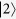. These processes give rise to *population correlations* of the two excited states. By contrast, 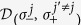 and 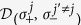 describe *cross-correlations* between the two transitions, allowing for bath-induced quanta exchange between the two excited states and thereby generating coherences between these states. Thus, the effect of the degeneracy is to mix the diagonal and the off-diagonal terms, via the cross-correlations in Eq. (9). We note that the evolution of this degenerate system is governed by a well-established master equation (see supplemental material)^34^,^35^,^39^,^41^,^42^,^43^.

A key parameter in the ensuing analysis is the dipole-alignment factor


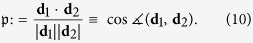


## Analysis

The energy that is continuously exchanged between the three-level system and the heat baths is related, according to the first law, to the power (the rate of work *W* extracted by the piston) by^44^


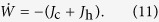


This expression involves the sum of heat currents from both baths, which can be derived from the dynamical version of the second law^2^. Their explicit expression for the *i*th bath (*i* ∈ {c, h}) is 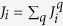, where the heat current 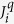 for the *q*th harmonic “sub-bath” (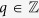) in Eq. (9) reads^2^,^37^


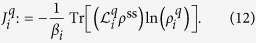


Here, 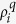 denotes the *local* steady state for a *single* heat bath at temperature *T*_*i*_ evaluated at the sideband *ω*_0_ + *q*Ω, i.e., 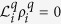. We stress that the *global* steady state *ρ*^ss^ (fulfilling 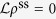) ensures the correct description of heat transport in this correlated three-state system, avoiding inconsistencies with the second law due to the improper use of local variables, as discussed in^45^. Since every Liouvillian 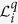 in the master equation (8) has the same functional dependence (9) on the atomic operators, the correct global solution can be directly obtained from the local one.

We here search for the steady-state solution of the master equation (8) and the resulting expressions for *J*_h(c)_. At this point we still do not know the bound for these currents and its dependence on alignment. These heat currents are therefore compared to the corresponding expressions (5)–(6) for a two-level system (TLS) with the same transition-dipole strength *d* and modulated transition frequency *ω*_0_ + *ω*(*t*)^21^.

The master equation (8) can be reduced to an analytically solvable inhomogeneous system of linear differential equations


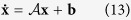


for the vector of matrix elements


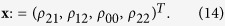


This system of ordinary differential equations (ODEs), where the matrix 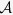 and the vector **b** are defined in Eqs. (31) and (32) in the Methods section, describes two very distinct dynamical regimes corresponding to aligned and misaligned transition dipoles, as detailed in what follows.

(i) Let us first consider the very general steady-state regime obtained for *misaligned transition dipoles*, 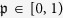. Note that this regime also includes the case of orthogonal dipoles (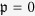). The three-level system then thermalizes to the diagonal steady state (without coherences)


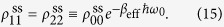



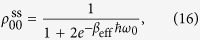


with an effective inverse temperature *β*_eff_ defined by the Boltzmann factor


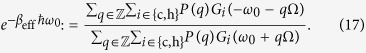


**Figure 2:**
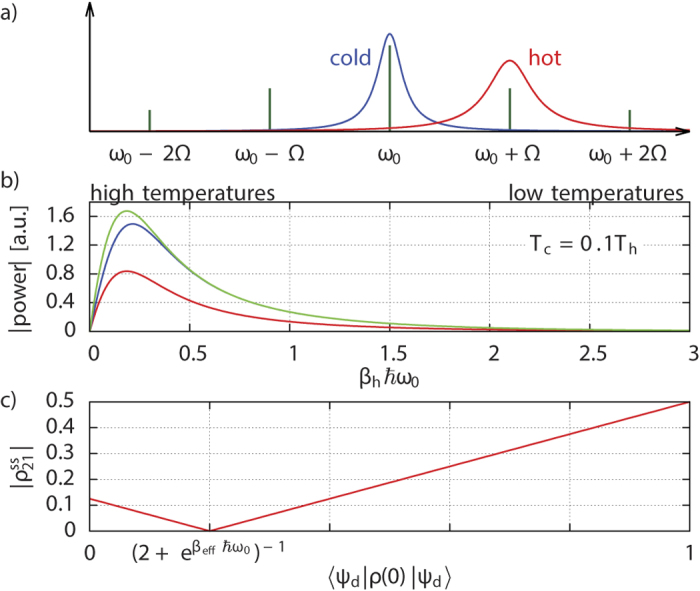
(**a**) “Engineering” of the effective temperature *T*_eff_ by controlling the weights of harmonic sidebands (via the modulation) in the two bath spectra. (**b**) Absolute value of the maximum power extraction (from bottom to top: TLS, non-aligned three-level system, aligned three-level system under optimal initial conditions) for *T*_c_ = 0.1*T*_h_. (**c**) Modulus 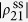 of the steady-state coherence for parallel dipoles. Maximal power boost [occurring for zero initial dark-state population according to Eq. (26)] corresponds to relatively small steady-state coherences. The highest steady-state coherence is realized for a dark initial state, which yields zero power.

This effective temperature determines the steady-state populations of the periodically modulated system coupled to both baths. We can control *β*_eff_ by engineering the modulation Floquet coefficients *P*(*q*) that determine the overlap of the sideband peaks (*q* = ±1, ±2,…) at the frequency harmonics *ω*_0_ + *q*Ω with the response spectra *G*_*i*_(*ω*) of the two baths, as sketched in Fig. 2a.

Upon computing the heat currents (12), we find that *J*_h_, *J*_c_ and the power 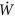 are modified (relative to their TLS counterparts in Eqs. (5) and (6)^21^) by the *same factor*


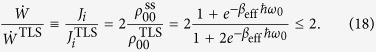


This means that the power enhancement relative to a TLS heat machine is determined by the ratio of the steady-state ground-state population 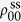 in the *V*-system to its TLS counterpart. Namely, in this fully thermalized incoherent regime the enhancement factor (18) only depends on the effective temperature (17).

(ii) For fully degenerate excited states we find that the coefficient matrix (31) of the ODE above is singular (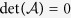) for *aligned* dipole moments (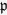 = 1). The same result holds for anti-parallel dipoles, which justifies the restriction of 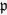 to non-negative values. This singularity implies that an infinite number of steady-state solutions may exist. Indeed, in this regime the dynamics is constrained by the existence of a dark state 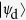, for which


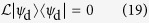



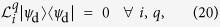


which renders the steady-state solution dependent on the initial conditions (in agreement with the expressions found for a single bath in refs. 34 and 46). The steady-state solution now depends on the overlap of the initial state *ρ*(0) with the non-dark states (i.e., the ground state 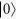 and the bright state 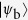) of the full Liouvillian 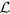 in Eqs. (8) and (9). The rôle of these states becomes apparent upon diagonalizing the steady-state solution, which yields the populations


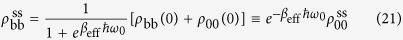



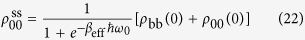



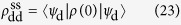


in the basis spanned by 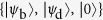. Here


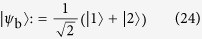


and


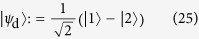


denote the bright and dark states, respectively. Whilst the dark-state population cannot change, i.e., it is a constant of motion (consistent with the one obtained in^35^ for a single zero-temperature bath and external driving), the bright and ground-state populations, *ρ*_bb_ and *ρ*_00_, respectively, thermalize. The same results hold for anti-parallel dipoles (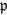 = −1) upon interchanging the dark and the bright states.

Proceeding as before in the non-aligned case, we find the power ratio


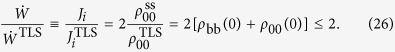


Hence, the power as well as the heat currents are enhanced in the aligned regime relative to their TLS counterparts by at most a factor of two, just as in the misaligned regime [Eq. (18)]. Yet, contrary to the latter, the ratio (26) does *not* depend on the bath spectra or the environmental temperatures, but solely on the initial populations of the non-dark states. Enhancement in Eq. (26) requires 
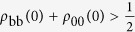
, or, equivalently, 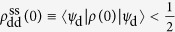, i.e., at least half of the initial-state population has to be non-dark. Maximal enhancement occurs when the initial state is amenable to full thermalization, i.e., it is non-dark.

For a given initial ground-state population *ρ*_00_(0), the states providing the maximum possible power boost are characterized by 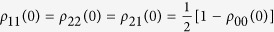. These are the states with the maximally allowed modulus of the *ρ*_21_(0) coherence (for a fixed ground-state population) and the correct phase. We have plotted the maximum power output under sinusoidal modulation for a TLS, a non-aligned, and an aligned *V*-system in Fig. 2b. The spectra are chosen as in ref. 21 such that only *G*_c_(*ω*_0_) and *G*_h_(*ω*_0_ + Ω) contribute (as sketched in Fig. 2a) and the modulation frequency has been tuned to the value maximizing the power output.

We stress that a non-dark initial state does not correspond to a steady state with maximal coherence 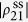 when rotating Eq. (21) back to the original basis spanned by 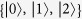. In fact, the coherence 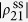 is maximized for an initial dark state, which does not exchange energy with the baths and gives zero power, see Fig. 2c.

It is natural to ask: How much initial overlap with the dark state is allowed such that the aligned configuration still outperforms its misaligned counterpart? The answer is, for


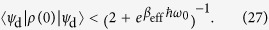


The value on the r.h.s. is the initial overlap for which the steady-state coherences vanish in the aligned case (see Fig. 2c).

So far we have made the comparison between the heat currents and the power, respectively, obtained for a three-level system relative to a two-level system. We now strive for a direct comparison of the enhancement factors (18) and (26) for the misaligned (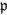 < 1) and aligned (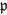 = 1) regimes. Their ratio is determined by the respective steady-state populations in the ground state, which is directly related to the power or heat-current ratio via


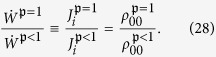


We consider this ratio in two limiting cases (assuming no initial overlap with the dark state in the aligned case):

(i) As *β*_eff_ → 0 (high effective temperature) the thermalized state corresponds to equipartition amongst the levels. For parallel dipoles, the thermalized three-level system behaves as a TLS (formed by the ground and the bright states) with an effective dipolar transition enhanced by the number of thermalization pathways, in this case two. Hence, in steady state half of the population is found in level 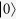 (if the initial state had no dark component). For misaligned dipoles, by contrast, thermal equilibrium corresponds to the equipartition amongst the three levels 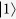, 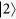 and 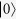. Consequently, only a third of the population is found in the ground state. The 3/2 ratio of the respective ground-state populations according to Eq. (28) explains the ratio of the maximal enhancement factors in the aligned and misaligned regimes at high *T*_eff_.

(ii) For large *β*_eff_, i.e., low *T*_eff_, however, Eq. (28) implies that the maximal enhancement for misaligned dipoles coincides with its counterpart for aligned dipoles (the latter is maximized for an initial state perpendicular to the dark state), since only 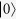 is then appreciably populated in either regime.

**Figure 3:**
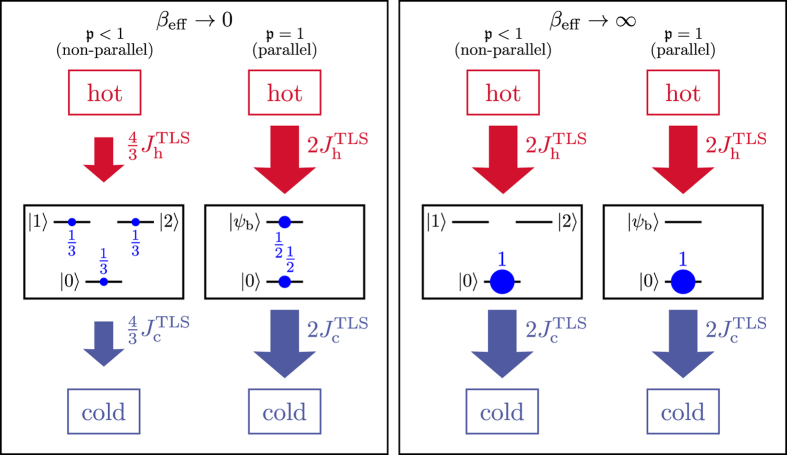
Limiting regimes of the heat currents for high (left panel) and low (right panel) effective temperatures. In case of parallel dipoles (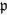 = 1) we assume an initial state orthogonal to the dark state to ensure maximal thermalization capability. The heat currents are related to the steady-state population of the ground state via Eq. (28).

Both regimes still retain the maximal enhancement factor of 2, stemming from their double thermalization pathways instead of one for a genuine TLS. We have summarized these results in Fig. 3. A beneficial influence of alignment on power output is only expected for effective temperatures 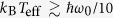. For optical transitions this corresponds to a few hundred Kelvin, whereas for microwave transitions the benefit of alignment is already expected for a few hundred milli-Kelvin.

## Realization considerations

*V*-systems with degenerate upper states are commonly found in atoms free of hyperfine interactions, e.g., mercury (Hg) or hydrogen (H). In particular, the three transitions 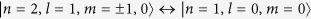 in such atoms are degenerate but have orthogonal transition dipoles. However, even such misalignment (orthogonality) does not hamper the *V*-system power boost at low *T*_eff_ (see above). The simultaneous coupling of such systems to hot and cold baths with controlled spectra can realize the misaligned case.

**Figure 4:**
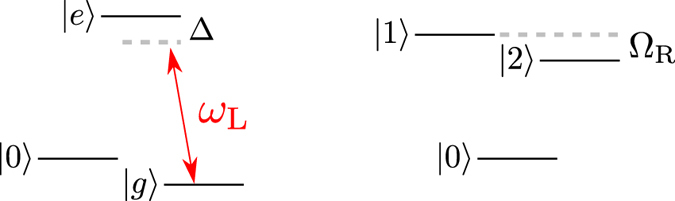
**Scheme suggested in ref.**^46^. The 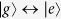 transition in a Λ-system is off-resonantly driven (left) to yield an effective nearly-degenerate *V*-system formed by the dressed states 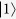 and 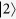 (right) with parallel transition-dipole momentes.

The case of degenerate upper states and parallel transition dipoles (which, as discussed, is beneficial for power enhancement *only at high T*_eff_), is obtainable only for transitions between a lower state with angular momentum *l* and magnetic number *m* and degenerate upper states with the same *m*^46^. In atomic degenerate *V*-systems such parallel transition dipoles are forbidden by selection rules. However, dressed states stemming from driven Λ-systems may effectively realize such parallel *V*-systems^46^ (see Fig. 4). Unfortunately, if we examine this system more closely, we see that it presents several difficulties: (i) The resulting transitions between the excited state doublet


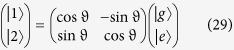


and the ground state, where *ϑ* is the mixing angle determined by the Rabi frequency Ω_R_ of the splitting field^46^, occur at rates that scale with 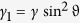 and 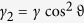, where *γ* is the decay rate of the bare excited state 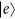. For maximal splitting (*ϑ* = *π*/4), *γ*_1_ = *γ*_2_ = *γ*/2. Hence the power boost is canceled by the reduction of the decay rate. (ii) In order to periodically modulate the transition frequency, we need an auxiliary field that induces an ac Stark shift only on the ground state. (iii) The dressed states are non-degenerate, which limits our results to time scales shorter than the inverse level splitting 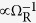. The latter, however, can be longer than the experimental time scale.

Molecules may be a more promising possibility due to their rich level structure involving rotational and vibrational degrees of freedom, as discussed in (See supplementary information in ref. 47).

## Discussion

Regardless of the transition-dipole misalignment or alignment, the maximally enhanced power output of a degenerate *V*-system heat engine is that generated by two independent two-level systems. The key to enhancement is the system to have degenerate upper levels sharing a *common ground state*. Hence, level degeneracy is found to be a thermodynamic resource that may effectively boost the power output. Yet, it does not affect the efficiency: Since the same modifying factors [Eqs. (18) and (26)] are obtained for the heat currents and the power, the efficiency


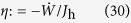


of the degenerate three-level heat machine is the same as for a two-level system. Thus, the same universal dependence of the efficiency on the modulation rate found in ref. 21 holds for the present system. In particular, as the heat currents (12) (by construction) fulfill the second and the first laws, they adhere to the Carnot bound^26^.

As shown in refs. 28 and 29, the efficiency of a continuous-cycle heat engine based on a TLS coupled to a quantized harmonic-oscillator “piston” is determined by the effective temperature and entropy-production rate of the piston: This efficiency may surpass the standard Carnot bound over many cycles if the piston is initially prepared in a small-amplitude coherent state. It is therefore possible that the extension of this model to a *V*-system may allow not only for a power boost but also an efficiency higher than the Carnot bound.

At effective temperatures significantly larger than the level spacing, the aligned-dipoles regime, where steady-state coherences arise, can outperform all misaligned cases. On the other hand, as discussed here, aligned transition dipoles can only be realized in a field-dressed atom, but such dressing divides the transition-dipole strength of the bare atom between two dressed-state transitions, and thereby cancels the power boost of the dressed-atom machine compared to its bare-atom counterpart.

This limitation of field-dressed atoms prompts the need for an alternative realization of aligned dipoles, free of such limitations, e.g., in molecules. Let us, however, assume that such a scheme can be realized and focus on conditions under which the aligned regime is advantageous in terms of its power boost compared to a TLS. We may not attribute the power enhancement to steady-state (or initial) coherences between the excited states but rather to the ability of the initial state to completely thermalize. We therefore conclude that (initially induced or steady-state) coherences are not an essential asset in the considered three-level-based heat machine. The existence of two thermalization pathways sharing a common ground state, regardless of whether they are coherent or incoherent, suffices for power enhancement.

## Methods

The coefficients of the ODE 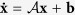 for **x**: = (*ρ*_21_, *ρ*_12_, *ρ*_00_, *ρ*_22_)^*T*^ read


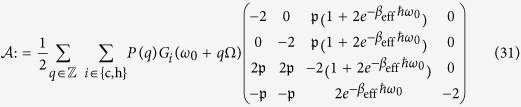


and


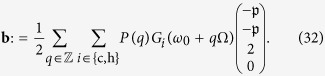


Note that the coherences between the ground and the excited states (*ρ*_10_ and *ρ*_20_) do not appear as they follow a decoupled dynamics (leading to vanishing steady-state values).

## Additional Information

**How to cite this article**: Gelbwaser-Klimovsky, D. *et al.* Power enhancement of heat engines via correlated thermalization in a three-level “working fluid”. *Sci. Rep.* **5**, 14413; doi: 10.1038/srep14413 (2015).

## Supplementary Material

Supplementary Information

## Figures and Tables

**Figure 1:**
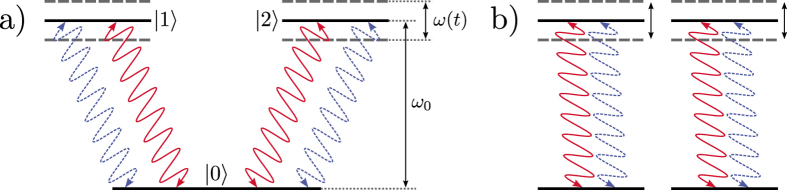
(**a**) A heat engine based on a degenerate *V*-type three-level system whose upper levels undergo periodic modulation while simultaneously interacting with a cold and a hot bath. The ground and excited states are incoherently populated by absorption of quanta from and (spontaneous and stimulated) emission to these baths (dotted blue: cold bath, solid red: hot bath). (**b**) Comparison to an analogous engine based on two independent two-level systems subject to the same environment and modulation.
