# Supplementary material for: Power enhancement of heat engines via correlated thermalization in a three-level “working fluid”
Source: Sci Rep. 2015 Sep 23;5:14413. doi: 10.1038/srep14413 (PMC4585770; doi:10.1038/srep14413)
Supplement: Supplementary Information [file srep14413-s1.pdf]

Supplemental material to: Power enhancement of heat engines via correlated thermalization in a three-level "working fluid"

David Gelbwaser-Klimovsky,[1,2] Wolfgang Niedenzu,[2] Paul Brumer,[3] and Gershon Kurizki[2]

[1]*Department of Chemistry and Chemical Biology, Harvard University, Cambridge, MA 02138, USA*

[2]*Department of Chemical Physics, Weizmann Institute of Science, Rehovot 7610001, Israel*

[3]*Chemical Physics Theory Group, Department of Chemistry and Centre for Quantum Information and Quantum Control, University of Toronto, Ontario M5S 3H6, Canada*

DERIVATION OF THE MASTER EQUATION ALLOWING FOR DEGENERACY OF THE "WORKING FLUID"

We consider a working fluid (system S) that is weakly coupled to a bath B, such that the total Hamiltonian reads

$$H = H_\mathrm{S} + H_\mathrm{B} + H_\mathrm{SB}, \tag{1}$$

where the last term is the system-bath interaction Hamiltonian. In the weak-coupling limit the Markovian master equation for the reduced density matrix of the system in the interaction picture reads ($\hbar = 1$) [1–4]

$$\frac{\mathrm{d}}{\mathrm{d}t}\rho(t) = -\int_0^\infty \mathrm{d}s\, \mathrm{Tr}_\mathrm{B}\left[H_\mathrm{SB}(t), \left[H_\mathrm{SB}(t-s), \rho(t)\otimes\rho_\mathrm{B}\right]\right], \tag{2}$$

where ρ_B is the state of the bath. The interaction Hamiltonian is of the form [1]

$$H_\mathrm{SB} = \sum_\alpha A_\alpha \otimes B_\alpha \tag{3}$$

with self-adjoint system and bath operators A_α and B_α, respectively. In particular, α may label the Cartesian coordinates $\alpha = x, y, z$ of the system and bath operators [1].

We next define the projector $\Pi(\varepsilon)$ onto the eigenspace associated to each energy eigenvalue ε of the system Hamiltonian. These eigenvalues can be either degenerate or non-degenerate [4]. The only rôle of degeneracy is to define the dimensionality of the subspace on which $\Pi(\varepsilon)$ acts. With the help of these projectors we decompose the system operators as [1, 4]

$$A_\alpha(\omega) = \sum_{\varepsilon'-\varepsilon=\omega} \Pi(\varepsilon) A_\alpha \Pi(\varepsilon'), \tag{4}$$

where ω are the discrete transition (Bohr) frequencies of the system. Transforming the interaction Hamiltonian (3) under this decomposition into the interaction picture [1], i.e.,

$$H_\mathrm{SB}(t) = \sum_{\alpha,\omega} e^{-i\omega t} A_\alpha(\omega)\otimes B_\alpha(t), \tag{5}$$

and inserting the result into the master equation (2) yields [1, 4]

$$\frac{\mathrm{d}}{\mathrm{d}t}\rho(t) = \sum_{\omega,\omega'}\sum_{\alpha,\beta} e^{i(\omega-\omega')t}\Gamma_{\alpha\beta}(\omega)\times \\ \times\left[A_\beta(\omega)\rho(t)A_\alpha^\dagger(\omega') - A_\alpha^\dagger(\omega')A_\beta(\omega)\rho(t)\right] + \mathrm{H.c.} \tag{6}$$

with the coefficients

$$\Gamma_{\alpha\beta}(\omega) := \int_0^\infty \mathrm{d}s e^{i\omega s}\left\langle B_\alpha^\dagger(t) B_\beta(t-s)\right\rangle. \tag{7}$$

For stationary baths the autocorrelation functions fulfill

$$\left\langle B_\alpha^\dagger(t) B_\beta(s-t)\right\rangle = \left\langle B_\alpha^\dagger(s) B_\beta(0)\right\rangle, \tag{8}$$

such that the coefficients $\Gamma_{\alpha\beta}(\omega)$ do not depend on time [1].

At this point we need to take into account the typical time scale on which the non-secular factors $e^{i(\omega-\omega')t}$ for $\omega\neq\omega'$ in the master equation (6) oscillate. If these terms oscillate very fast during the time the system density operator is evolving, these oscillations will average out, allowing us to neglect the non-secular terms $\omega\neq\omega'$ (corresponding to the secular approximation) [1]. The resulting master equation is then valid at times

$$t \gg \max\frac{1}{\omega-\omega'} \quad (\omega\neq\omega'). \tag{9}$$

We stress that the possible degeneracy of the energy eigenvalues ε and ε' allowed for in Eq. (4) is irrelevant to condition (9), which involves discrete transition (Bohr) frequencies ω and ω' in the Fourier decomposition of the system operators (5) and the master equation (6).

By this procedure one finds the Liouvillian superoperator [1]

$$\mathcal{L}\rho = \sum_\omega\sum_{\alpha,\beta}\frac{1}{2}\gamma_{\alpha\beta}(\omega)\times \\ \times\Big(2A_\beta(\omega)\rho A_\alpha^\dagger(\omega) - A_\alpha^\dagger(\omega)A_\beta(\omega)\rho - \rho A_\alpha^\dagger(\omega)A_\beta(\omega)\Big) \tag{10}$$

with the rates

$$\gamma_{\alpha\beta}(\omega) = \Gamma_{\alpha\beta}(\omega) + \Gamma_{\beta\alpha}^*(\omega) = \\ = \int_{-\infty}^\infty \mathrm{d}s e^{i\omega s}\left\langle B_\alpha^\dagger(s)B_\beta(0)\right\rangle. \tag{11}$$

Assuming dipolar coupling of the atoms to the bath, i.e., an interaction Hamiltonian of the form

$$H_{\mathrm{SB}} = \mathbf{D} \cdot \mathbf{B}, \tag{12}$$

following the lines of Sec. 3.4. of Ref. [1] and allowing for the periodic time dependence of the system Hamiltonian (Eq. (1) in the main text) as in Refs. [5, 6], we then find the master equation (9) of the main text. Therein we have made use of the projectors

$$\Pi(0) = |0\rangle\langle 0| \tag{13}$$

onto the ground state and

$$\Pi(\omega_0) = |1\rangle\langle 1| + |2\rangle\langle 2| \tag{14}$$

onto the doubly-degenerate excited-state subspace. These projectors yield the decomposition of the Cartesian coordinates $\alpha = x, y, z$ of the dipole operator $\mathbf{D}$ [as in Eq (4)],

$$A_\alpha(\omega_0) = \Pi(0) D_\alpha \Pi(\omega_0) = (d_1)_\alpha \sigma_-^1 + (d_2)_\alpha \sigma_-^2, \tag{15}$$

with $(d_k)_\alpha := \langle 0|D_\alpha|k\rangle$ for $k \in \{1, 2\}$.

[1] H.-P. Breuer and F. Petruccione, *The Theory of Open Quantum Systems* (Oxford University Press, 2002).

[2] H. Carmichael, *An Open Systems Approach to Quantum Optics* (Springer-Verlag, Berlin Heidelberg, 1993).

[3] C. W. Gardiner and P. Zoller, *Quantum Noise*, 2nd ed. (Springer-Verlag, Berlin, 2000).

[4] S. Kryszewski and J. Czechowska-Kryszk, arXiv preprint arXiv:0801.1757 (2008).

[5] R. Alicki, D. Gelbwaser-Klimovsky, and G. Kurizki, arXiv preprint arXiv:1205.4552 (2012).

[6] R. Kosloff, Entropy **15**, 2100 (2013).
